# Insights from mathematical modeling for convection-enhanced intraputamenal delivery of GDNF

**DOI:** 10.1007/s11517-017-1650-x

**Published:** 2017-05-11

**Authors:** Elena Belova, Christopher L. Shaffer, Patrick E. Trapa

**Affiliations:** Worldwide Research & Development, Pfizer Inc., 1 Portland Street, Cambridge, MA 02139 USA

**Keywords:** Convection-enhanced delivery (CED), Central nervous system (CNS), Neurotrophic factor, Intraparenchymal infusion, Computational model

## Abstract

Glial cell line-derived neurotrophic factor (GDNF) is a potential therapy for Parkinson’s disease (PD) promoting survival and functional recovery of dopaminergic neurons when delivered to the degenerated striatum. To study the aspects of intraputamenal delivery of GDNF, a mathematical model of recombinant methionyl human GDNF (r-metHuGDNF) convection in the human putamen has been developed. The convection-enhanced delivery infusions of r-metHuGDNF were simulated at rates up to 5 μL/min. The high-rate infusions (≥1 μL/min) permit rapid and uniform distribution of drug with up to 75% of the distribution volume having a concentration within 5% of the infusate concentration. No relevant differences in distribution at infusion rates of 3 and 5 μL/min were found. The patterns of GDNF distribution were analyzed in relation to the anatomy of the posterior dorsal putamen, and a cylindrical shape was found to be preferable considering risks of target overflow. A magnetic resonance (MR) tracer Gd-DTPA (Magnevist®) was evaluated as a surrogate in clinical studies, and the most accurate prediction of GDNF distribution was calculated immediately after infusion. The clearance of GDNF from the striatum is confirmed to be slow, with a half-life of ca. 19 h.

## Introduction

Intraparenchymal convection-enhanced delivery (CED) was first introduced in 1994 to overcome the blood-brain barrier (BBB) and to enhance drug distribution in the central nervous system [1]. The CED technique relies on convection established by a pressure gradient at the tip of the infusion catheter. The bulk flow augments diffusion and produces more widespread and uniform distribution of drug molecules in the brain. The CED infusion rate should be high enough to induce convection but low enough to be safely tolerated by patients. Drug distribution volume (*V*_d_) is directly proportional to the infusion volume (*V*_i_) with a ratio that is specific to the tissue type [2]. After cessation of CED, the drug continues to spread by diffusion alone until it is cleared from the tissue. One of the challenges for CED is reflux or backflow of infusate along the catheter track which can lead to poor coverage as well as possible adverse events (AEs) stemming from exposures outside of the region of interest. Loss due to reflux can reach up to 50% of the total *V*_d_ in high-volume infusions [3]. Modern CED platforms demonstrate the ability to minimize reflux while achieving large *V*_d_ in the target [4].

Glial cell line-derived neurotrophic factor (GDNF) [5] was originally isolated from a rat glioma cell line and identified as a growth factor promoting the survival of dopaminergic (DA) neurons. It has been examined as a drug candidate for the treatment of PD, which is marked by progressive loss of nigrostriatal DA neurons. Studies with rodent and non-human primate models of PD have confirmed the neurorestorative and neuroprotective effects of GDNF when delivered into the cerebral ventricles or directly into the striatum or substantia nigra [6–9]. Preclinical findings led to several trials in PD patients. In the first study, recombinant methionyl human GDNF (r-metHuGDNF) was administered intracerebroventricularly (ICV) to patients by monthly bolus injections. The study failed to provide clinical benefits but demonstrated significant AEs [10]. A postmortem examination of one patient revealed that the GDNF ICV treatment did not induce regeneration of nigrostriatal neurons in this patient [11]. To achieve efficacy and reduce AEs, three subsequent trials delivered r-metHuGDNF directly into the putamen of PD patients via continuous infusion driven by intra-abdominal pumps. Two small open-label studies reported substantial improvements in patient motor function with adequate safety and tolerability after 6–12 months of infusion [12, 13]. However, a third randomized double-blind placebo-controlled trial in patients with moderate-to-severe PD did not detect a significant improvement in motor outcome compared with placebo despite a modest local increase in [^18^F]DOPA uptake as shown by positron emission tomography (PET) [14]. Retrospectively, the limited distribution of r-metHuGDNF caused by technical challenges was hypothesized to be partly responsible for trial failure [15]. Since patient *V*_d_ were not assessed throughout the trial, the clinical performance of the infusion system was not evaluated.

More recently, a randomized double-blind placebo-controlled trial was designed to explore the benefits of a chronic intermittent dosing regimen for intraputamenal (IPu) delivery [16, 17]. Consistent with the expectation that this regimen would increase coverage of the striatum and prevent off-target distribution [18], the study required a distinct minimum coverage of a predefined putamenal volume of interest for patients to proceed to randomization [17]. Intermittent administration was supported by observations in rats of prolonged neurorestorative effects of GDNF in the striatum after a single intrastriatal infusion [19, 20]. The aim of the present work was to model prospectively the distribution of r-metHuGDNF in the putamen following a short-term IPu infusion.

Our model was built on the framework introduced by Morrison et al. [21] in 1994 to mathematically describe transport of macromolecules in intraparenchymal CED. The authors used a rigid-pore assumption to model brain tissue and applied the convection-diffusion-reaction differential mass balance equation to define macromolecular transport. Their theory was broadly adopted for CED modeling, where computational results are usually validated in preclinical models [21–23]. The final structure of the model depends on the CED application, namely a microstructure of the target, properties of the drug, parameters of the CED platform, and delivery protocol.

## Methods

### Mathematical model

**Fig. 1:**
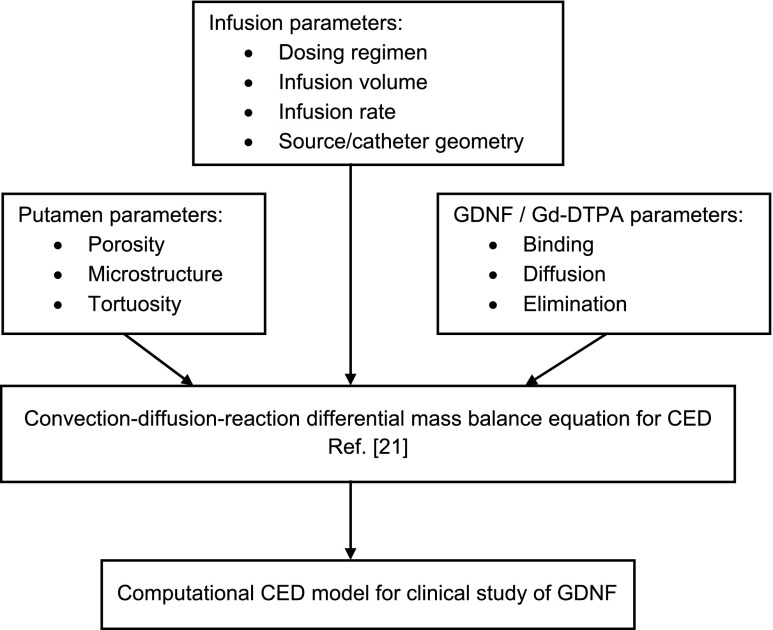
Schematic of the generation of the applied model from CED theory

The modeling approach is illustrated in Fig. 1. The general convection-diffusion-reaction differential mass balance equation for CED was defined in Morrison et al. [21]. Applying it to the transport of r-metHuGDNF in the putamen, we neglected macromolecular binding, both specific and non-specific, in interstitial fluid (ISF). Our assumption was supported by studies in non-human primates (NHPs), where it was found that in IPu CED the difference in *V*_d_ between GDNF and the MRI tracer gadoteridol (Gd) was slight [24], suggesting the negligible role of binding in the distribution process. The next assumption was to ignore the loss of GDNF across the capillary walls because of the low ISF flow rate. As it will be shown in Section 3, in the present model, the infusion times necessary to simulate *V*_d_ were about 1–2 h depending on the CED rate, while an ISF turnover time is about 20 h [25]. With these assumptions, the transport of non-binding macromolecules is governed by the equation

1 $$ \phi \frac{\partial {C}_{\mathrm{ISF}}}{\partial t}=\nabla \cdot \left(\phi {\mathbf{D}}_{\mathbf{t}}\cdot \nabla {C}_{\mathrm{ISF}}\right)-\nabla \cdot \left(\phi {\overrightarrow{v}}_{\mathrm{ISF}}{C}_{\mathrm{ISF}}\right)-{k}_{\mathrm{irr}}{C}_{\mathrm{ISF}}, $$

where *C*_ISF_ is the concentration of r-metHuGDNF or Gd-DTPA in ISF, *ϕ* is the tissue porosity, ${\overrightarrow{v}}_{\mathrm{ISF}}$ is the interstitial velocity; **D**_**t**_ is the macromolecular diffusion tensor in the putamen, and *k*_irr_ is a first-order degradation rate constant [2, 21].

The diffusion tensor is defined by the regional microstructure of the target. The fractional anisotropy (FA) of the putamen was measured in diffusion tensor imaging (DTI) studies in healthy elderly subjects and patients with mild-to-moderate PD [26]. In almost all cases, the reported FA was below an approximate threshold value for the gray matter (FA < 0.275) defined by Kim et al. [22]. The latter means an isotropic distribution of macromolecules within the putamen with a constant value for diffusivity [27]:

2 $$ {D}_t=\frac{D}{\lambda^2}, $$

where *D* is the diffusion coefficient in the free medium and *λ* is the tortuosity characterizing the hindrance to diffusion in brain ISF.

To define the interstitial fluid velocity ${\overrightarrow{v}}_{\mathrm{ISF}}$, we solved the equation for continuity of brain ISF:

3 $$ \nabla \cdot {\overrightarrow{v}}_{\mathrm{ISF}}=0, $$

where 0 in the right part of the equation is consistent with our assumption to ignore ISF exchange in the simulations. Assuming a spherical symmetry of distribution from the catheter in the isotropic target and given the boundary condition at the catheter tip [28],

4 $$ Q=4\pi {r_0}^2\phi {v}_r, $$

where *Q* is the volumetric infusion rate and *r*_0_ is the radius of the catheter tip; the solution for the radial interstitial velocity is

5 $$ {v}_r=\frac{Q}{4\pi \phi {r}^2}, $$

where *r* is the distance from the catheter tip, *r* > *r*_0_.

Thus, the final model equation defining the transport of either non-binding r-metHuGDNF or Gd-DTPA in the putamen is

6 $$ \frac{\partial {C}_{\mathrm{ISF}}}{\partial t}=\frac{D}{\lambda^2}\frac{1}{r^2}\frac{\partial }{\partial r}\left({r}^2\frac{\partial {C}_{\mathrm{ISF}}}{\partial r}\right)-{v}_r\frac{\partial {C}_{\mathrm{ISF}}}{\partial r}-{kC}_{\mathrm{ISF}}, $$

where *k* represents a first-order elimination rate constant assumption and was calculated from the preclinical studies for recombinant human GDNF [19]. Equation (6) was solved numerically in MATLAB R2010b (MathWorks, Natick, MA) using a finite element technique. For convenience, Eq. (6) was solved for dimensionless concentration *C*_norm_ via scaling the equation to the initial infusate concentration *C*_0_:

7 $$ {C}_{\mathrm{norm}}=\frac{C_{\mathrm{ISF}}}{C_0}. $$

### Elimination rate

Two approaches were used to determine the rate of elimination *k* in Eq. (6). First, the clearance rate was calculated from the published pharmacokinetic (PK) study of a single GDNF infusion into the rat striatum [19]. In that study, two doses of recombinant human GDNF (15 and 3 μg) were infused by CED, and GDNF concentration in the whole striatum was measured at 3, 7, 14, 21, and 28 days post-infusion. These data were fit with a two-compartment PK model in NONMEM v.7.2 (ICON Development Solutions, Hanover, MD), where the brain extracellular space was taken as the central compartment to estimate the rate constants, mean(SE): *k*_10_ = 3.72(0.44)×10^−2^ *h* ^−1^, *k*_12_ = 7.12(1.20)×10^−4^ *h* ^−1^, and *k*_21_ = 3.81(0.76)×10^−3^ *h* ^−1^. Since *k*_12_ <  < *k*_10_, then *k*_*el*_ ∼ *k*_10_, a first-order elimination is able to fit the GDNF concentration at 3 and 7 days after infusion, while the second exponent input (*k*_21_) becomes important at later time points. First-order elimination was therefore deemed sufficient to capture initial distribution, and the rate constant *k* = *k*_10_ was introduced into the model. The calculated half-life of GDNF in the striatum was approximately 19 h.

The elimination rate was also calculated using the ISF flow rate explicitly assuming that clearance by ISF flux is the main elimination pathway. If the ISF outflow rate is approximately 0.17 μL per gram of brain per minute [25], then for a typical rat brain weighing 1.8 g, the flow rate *Q*_ISF_ is 0.306 μL/min. The total ISF volume, 540 μL, was projected from the assumption of brain tissue porosity:

8 $$ \phi =\frac{V_{\mathrm{ISF}}}{V_{\mathrm{brain}}}, $$

where the *ϕ* value is between 0.2 and 0.4 [21]. Therefore, *ϕ* = 0.3 was used in the present work. Consequently, the elimination rate by ISF flux is

9 $$ k=\frac{Q_{\mathrm{ISF}}}{V_{\mathrm{ISF}}}, $$

where *k* = 3.40 ⋅ 10^−2^ *h* ^−1^. This rate was very close to the prediction from the rat PK study suggesting that the main mechanism of elimination of GDNF from the striatum is clearance by the slow ISF flow.

### Diffusion parameters

Diffusion parameters of r-metHuGDNF, a homodimer with a molecular weight of 30.4 kDa [20], were extrapolated from the diffusion study of a bioactive rhodamine nerve growth factor (NGF) conjugate (26.5 kDa) in the rat striatum [29, 30] using the Stokes-Einstein relation and a standard assumption of a spherical shape for the diffusing molecules: *D*_GDNF_ = 1.3 ⋅ 10^−6^cm^2^/s; *λ*_GDNF_ = 2.2. The diffusion coefficient of Gd-DTPA (Magnevist®, 938 Da) was extrapolated from the obtained *D*_GDNF_, explicitly assuming an inverse one-third power dependence of diffusivity on molecular weight: *D*_Gd ‐ DTPA_ = 4.1 ⋅ 10^−6^cm^2^/s. The Gd-DTPA tortuosity value, *λ*_Gd ‐ DTPA_ = 1.6, was chosen from the range of 1.5–1.6 measured for small molecules in brain ISF [30].

### Loss during infusion

To estimate the amount of drug cleared in the process of infusion, the following assumptions were made: (1) intraputamenal drug concentration remains unchanged during the short infusion and equals the infusate concentration, *C*_0_; (2) *V*_d_ is proportional to *V*_i_ with a constant ratio (*V*_d_/*V*_i_); (3) infusion occurs at a constant rate *Q*; (4) distribution caused by diffusion is negligible in CED; and (5) binding, both specific and non-specific, was ignored. Based on these assumptions, the differential mass balance equations are

10 $$ \frac{dA(t)}{dt}=- kA(t)+{QC}_0\left(\frac{V_{\mathrm{d}}}{V_{\mathrm{i}}}\right), $$

11 $$ \frac{dA_{\mathrm{Loss}}(t)}{dt}= kA(t), $$

where *A* is the total amount of drug while *A*_Loss_ is the amount cleared (i.e., lost), and initial conditions are

$$ \begin{array}{l} A(0)=0,\\ {}{A}_{\mathrm{Loss}}(0)=0.\end{array} $$

The equations were solved symbolically in MATLAB R2010b (MathWorks, Natick, MA), and the following solution for *A*_Loss_ was obtained:

12 $$ {A}_{\mathrm{Loss}}(t)={QC}_0\left(\frac{V_{\mathrm{d}}}{V_{\mathrm{i}}}\right)\left( t-\frac{1-{e}^{- kt}}{k}\right). $$

### Distribution in target

**Fig. 2:**
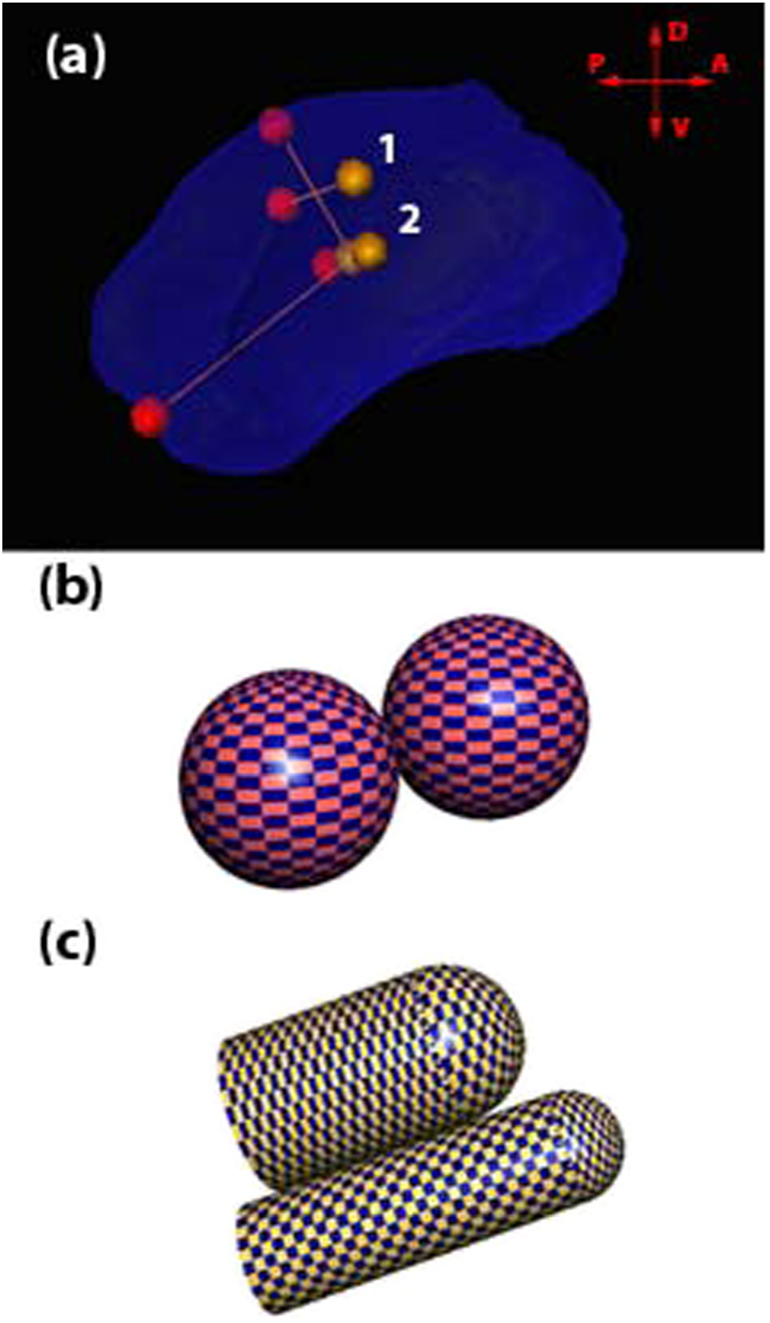
The left human putamen from the ICBM atlas with the outlined region of interest (ROI). Labels *1* and *2* indicate two measurements of ROI width. Orientation is indicated by *arrows*: *P* posterior, *D* dorsal, *A* anterior, *V* ventral (**a**). Distribution patterns for two catheters with isolated either spherical *V*_d_ (**b**) or cylindrical *V*_d_ (**c**)

In previous clinical trials, the target for CED infusions was the posterior dorsal putamen since this is the region most depleted of dopamine in PD [14]. We have projected *V*_d_ in this target using the measurements manually extracted from the ICBM (International Consortium for Brain Mapping) template atlas using Amira v.5.5 (FEI Visualization Sciences Group, Bordeaux, France) for the left posterior dorsal putamen: 1.92 cm (length), 1.15 cm (height), and 1.12 or 0.90 cm (width, the measurement depends on the location as shown in Fig. 2a). Reported MRI-based measurements suggest the putamen volume in PD patients is 3.98 ± 0.15 cm^3^ [31]. We assumed that the target is roughly 25% of total putamen and predicted a volume of interest of 1 mL per putamen. Since the employed CED system is proposed to utilize four microcatheters per patient [16], or two catheters per putamen, our estimation for *V*_*d*_ per catheter was 0.5 mL.

In isotropic tissues, a reflux-resistant CED catheter produces a uniform spherical *V*_d_ around the infusion site (Fig. 2b):

13 $$ {V}_{\mathrm{d}}=\frac{4}{3}\pi {r}^3, $$

where *r* is the distributional radius. Thus, for 0.5 mL *V*_d_, the corresponding distributional diameter is 0.98 cm (Table 1). In the model, the volume occupied by the catheter tip in the target was negligible compared to the total achieved *V*_d_. For example, a typical cannula with a length of 1 cm and an outer diameter of 1 mm [3] occupies a volume of about 8 μL.

**Table 1:** Anatomical parameters of the human posterior dorsal putamen and models of 0.5 mL *V*_d_

Dimensions	ICBM measurements^a^ (cm)	Spherical *V*_d_ model^b^ (cm)	Cylindrical *V*_d_ model^b^ (cm)
Length	1.92	0.98	0.70–1.90
Height	1.15	0.98	0.60–1.12
Width	1.12, 0.90^c^	0.98	0.60–1.12

^a^Measurements made for the left posterior dorsal putamen

^b^Calculations per catheter; each putamen contains two catheters

^c^Measured at two levels, labels 1 and 2 from Fig. 2a, respectively

Catheter design impacts the shape of the distribution. Cylinder-like *V*_d_ are more typical for conventional catheters because of reflux (i.e., backflow) [3]. When reflux is controlled and constrained within the target, the cylindrical distribution may be more suitable for non-spherical regions of interest. In this case, the total *V*_d_ can be estimated as a sum of two volumes, the hemisphere formed below the tip and the cylindrical volume refluxed above the tip (Fig. 2c):

14 $$ {V}_{\mathrm{d}}=\frac{2}{3}\pi {r}^3+\pi {r}^2 h, $$

where *h* is the reflux length and (*r* + *h*) is the total length. The total length and diameter are related inversely. For example, for projected 0.5 mL *V*_d_, two possible scenarios were calculated: 0.70 cm distributional length and 1.12 cm distributional diameter, or 1.90 cm distributional length and 0.60 cm diameter (Table 1). Also, in the case of cylindrical *V*_d_, the angle of catheter inclination becomes an additional parameter to optimize the volume.

## Results

### GDNF distribution at different infusion rates

Infusions were modeled at several possible rates. Simulation parameters are listed in Table 2. In the early GDNF PD trials, IPu infusions were continuous at a non-CED rate of 0.1 μL/min [12]. However, the minimal rate to induce convection is 0.5 μL/min [18]. A novel improved CED system can deliver reflux-free infusions at rates of up to 5 μL/min [3]. Safety of CED infusions has been assessed in preclinical and clinical studies to a reported rate limit of 10 μL/min [2]. Based on this, the following infusion rates were investigated: 0.1, 1, 3, and 5 μL/min.

**Table 2:** Simulation parameters

Parameter	Symbol	Value
Porosity	*ϕ*	0.3
Distribution volume (mL)	*V*_d_	0.5
Distribution distance (cm)	*r*	0.3 − 0.56
Source radius (cm)	*r* ^′^_0_	0.2
Infusion rate (μL/min)	*Q*	0.1 − 5
Infusion volume (mL)	*V*_i_	0.105
Diffusion coefficient (cm^2^/s)	*D*_GDNF_	1.3 × 10^−6^
	*D*_Gd ‐ DTPA_	4.1 × 10^−6^
Tortuosity	*λ*_GDNF_	2.2
	*λ*_Gd ‐ DTPA_	1.6
Elimination rate constant (h^−1^)	*k*	3.72 × 10^−2^

**Fig. 3:**
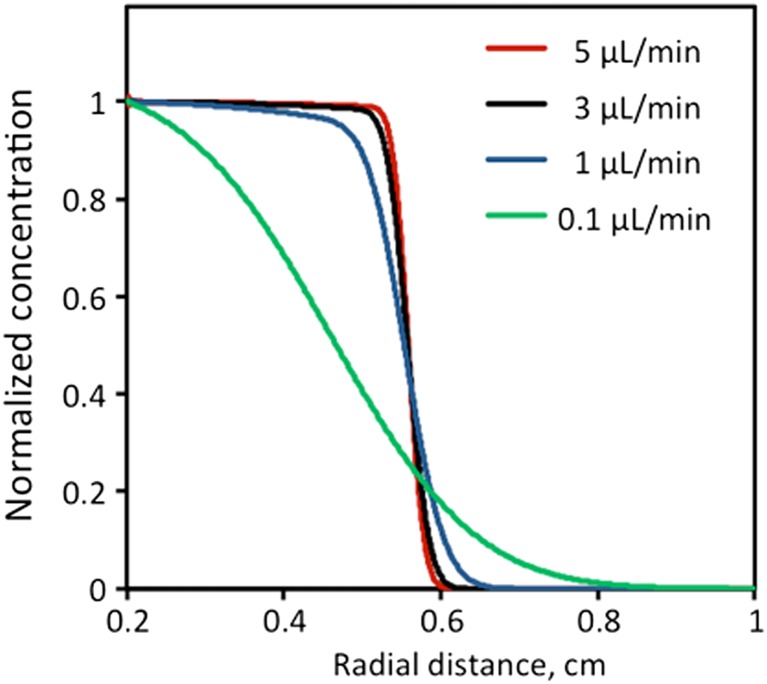
R-metHuGDNF concentration profiles developed at different infusion rates. All infusions were simulated from the 0.2-cm sphere and lasted until the total *V*_i_ of 0.105 mL was delivered

Concentration profiles have been simulated for all four rates (Fig. 3). Infusions at the highest rates, 3 and 5 μL/min, produce nearly identical concentration profiles. Considering the geometry of the target and estimations for *V*_d_ made beforehand (Table 1), the distribution of GDNF is expected within radial distances (*r*) of 0.30 to 0.56 cm from the catheter. Infusions were simulated from the sphere around the catheter tip at a radius of 0.2 cm (*r* ^′^_0_) to avoid numerical instability caused by very high instantaneous exit velocity *v*_*r*_ near the catheter tip (e.g., with a typical diameter of 0.06 cm) [3]. A *V*_i_ of 0.105 mL was chosen for simulation to reach *C*_norm_ = 0.5 at a distance of ca. 0.55 cm, although the profile for the lowest infusion rate of 0.1 μL/min broadened due to diffusion and *C*_norm_ = 0.5 was shifted to the left after the infusion (Table 3).

**Table 3:** R-metHuGDNF distribution parameters at different infusion rates

Rate (μL/min)	*C*_norm_ = 0.5		*V*_d_ (mL)		*V*_d_^0.95^/*V*_d_^0.05^
	*r* (cm)	*Pe*	*C*_norm_ ≤ 0.95	*C*_norm_ ≤ 0.05	
0.1	0.467	3.5	0.072	1.479	0.05
1	0.553	30	0.444	0.997	0.45
3	0.558	88	0.590	0.877	0.67
5	0.558	147	0.630	0.837	0.75

The Peclet number (*Pe*), which describes the ratio of mass transfer by convection to diffusion, was used to characterize the GDNF distribution at each infusion rate. Within the model, this ratio equals

15 $$ Pe=\frac{Q}{4\pi \phi \frac{D_{\mathrm{GDNF}}}{\lambda_{\mathrm{GDNF}}^2} r}. $$

*Pe* is decreased proportionally with the radial distance from the catheter *r*. The minimal *Pe* was calculated for each infusion rate at the distance associated with *C*_norm_ = 0.5.

To determine the homogeneity of distribution, a ratio of high-concentration coverage to the total *V*_d_ was calculated. The threshold values for the high-concentration *V*_d_ and total *V*_d_ were *C*_norm_ = 0.95 and *C*_norm_ = 0.05, respectively (Table 3). For the 1, 3, and 5 μL/min infusion rates, the high-concentration portion accounts for 45, 67, and 75% of total *V*_d_, respectively. Such uniform distribution occurs because bulk flow dominates over diffusion as characterized by *Pe* >  > 1. At 0.1 μL/min, the Peclet number is close to 1 and both processes, bulk flow and diffusion, impact the distribution. Diffusion not only broadens the coverage but also diminishes homogeneity, reducing the high-concentration portion to 5%.

The calculated *V*_d_ per catheter in this study is 0.5 mL. Assuming the linear dependence between the infusion and distribution volumes and a *V*_d_/*V*_i_ = 3.87 measured in the NHP putamen [24], the required infusion volume is projected as 0.13 mL per catheter. In the model, all high-rate infusions (≥1 μL/min) resulted in a uniform square-shaped concentration profile, a defining characteristic of convection-dominated transport. To deliver the projected *V*_i_, the infusion time is set as 130, 43, and 26 min for 1, 3, and 5 μL/min rates, respectively. Although the elimination of GDNF from the target is slow, some amount of the infusate is cleared over the course of the infusion. Assuming a constant concentration of infusate in the target (*C*_norm_ = 1), we have estimated the portion of *V*_d_ which is cleared during the infusion using Eq. (12). Calculated *V*_Loss_ were 0.020, 0.007, and 0.004 mL for infusions at 1, 3, and 5 μL/min, respectively. Greater loss occurs when the drug is infused at slower rates because the infusion time is longer. This effect was also observed in the simulations, when the 1 μL/min concentration profile, while still steep, had a slightly lower amplitude (*C*_norm_) than the other two profiles. No differences were found between the 3 and 5 μL/min rates; they produced nearly identical concentration profiles characterized by approximately 1% of *V*_d_ loss during the infusion phase.

### Correlation between Gd-DTPA and GDNF distributions

**Fig. 4:**
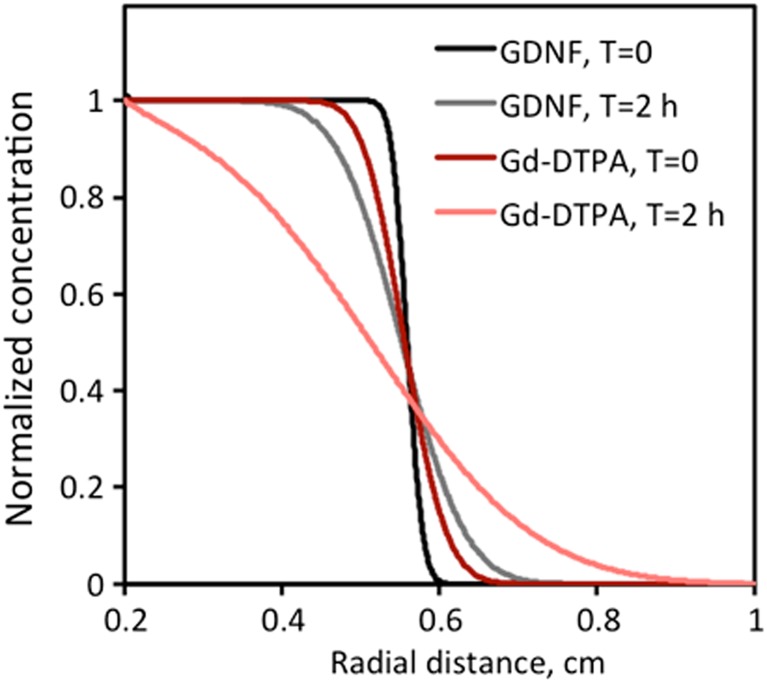
5 μL/min infusion concentration profiles of r-metHuGDNF (*black lines*) and Gd-DTPA (*red lines*) immediately (*T* = 0, *colorful lines*) and 2 h after (*T* = 2 h, *pale lines*)

The distribution of drug delivered via CED can be approximated by imaging test infusions of MRI-visible tracers, such as clinical-grade Gd-DTPA (Magnevist®) [32]; however, the transport properties of small and large molecules, in this case Gd-DTPA (<1 kDa) and r-metHuGDNF (ca. 30 kDa), differ. Simulations were performed to compare the distributions using an infusion rate of 5 μL/min (Fig. 4). Both infusions were again simulated from the sphere around the catheter with a radius of 0.2 cm (*r* ^′^_0_) and a *V*_i_ of 0.105 mL. Simulated concentration profiles were plotted immediately post-infusion and 2 h thereafter to illustrate the temporal dependence of Gd-DTPA and r-metHuGDNF distributions. Our previously described estimations of the GDNF elimination rate suggest that the main GDNF clearance mechanism appears to be ISF bulk flow; therefore, the elimination rate should not depend on the size of impermeable molecules. Thus, the elimination rate of Gd-DTPA would be expected to equal that of GDNF since neither cross the intact BBB. Consequently, the clearance process should not significantly affect the difference in distribution between the two compounds within the 2-h time frame, and hence, it was ignored in simulations.

To compare the Gd-DTPA and GDNF distributions, *V*_d_ for both molecules were calculated from the simulation using Eq. (13) and assuming a uniform spherical distribution in the target. The distributional radii for each *V*_d_ were defined at the tissue concentration threshold value, arbitrarily chosen as a 10% of *C*_0_ (*C*_norm_ = 0.1). Simulated *V*_d_ of GDNF were 0.81 mL immediately after the infusion was ended (*T* = 0) and 1.07 mL 2 later (*T* = 2 h). *V*_d_ of Gd-DTPA were 0.95 mL immediately after the infusion (*T* = 0) and 1.58 mL 2 h after the infusion (Table 4). Thus, within 2 h, the *V*_*d*_ increase was 1.3-fold for GDNF and 1.7-fold for Gd-DTPA. The correlation between Gd-DTPA and GDNF *V*_d_ was calculated as a *V*_d_^Gd ‐ DTPA^/*V*_d_^GDNF^ ratio and found to be 1.2 immediately after the infusion (*T* = 0) and 1.5 2 h later (*T* = 2 h).

**Table 4:** Correlation between r-metHuGDNF and Gd-DTPA distribution volumes (*V*_d_) immediately and 2 h after ending a 5 μL/min infusion

	*T* = 0	*T* = 2 h
*V*_d_^Gd ‐ DTPA^ (mL)	0.95	1.58
*V*_d_^GDNF^ (mL)	0.81	1.07
*V*_d_^Gd ‐ DTPA^/*V*_d_^GDNF^	1.2	1.5

The diffusion of molecules depends on their size with smaller molecules traveling faster and therefore farther in the tissue. The tortuosity values characterizing the hindrance to diffusion in brain ISF are also reduced for small molecules [30]. As a result, an effective diffusion coefficient of Gd-DTPA in the striatum, *D*_*t* , Gd ‐ DTPA_, defined in Eq. (2), was 6-fold greater than *D*_*t* , GDNF_; therefore, larger *V*_d_ were found in Gd-DTPA simulations. The more accurate prediction of r-metHuGDNF *V*_d_ was found during the infusion process, when the difference in diffusion was compensated by bulk flow.

## Discussion

Modeling suggests that short, intermittent, CED infusions afford the opportunity to optimize both the volume and the pattern of GDNF distribution. Such precise delivery is particularly beneficial considering the potential risks associated with exposing non-target structures to GDNF [18]. Recently, distribution shape has been tailored through modifications of the step length of a recessed-step catheter [4]. The present analysis, based on the anatomy of the human putamen, suggests that cylindrical patterns permit more flexibility in target coverage. Specifically, cylindrical distribution with custom height and width may be used to accommodate intersubject anatomical variability of target structures.

The present model is a preliminary approach to describe an IPu delivery of GDNF in patients. Further refinement requires clinical data. For example, the next step will be incorporating DTI data to project the degree of anisotropy and inhomogeneity of the putamen and surrounding tissue. The MR-DTI technique measures the effective tensor of water diffusion in the tissue which is sensitive to the tissue microstructure. The water diffusion tensor is then used to assign directionality to **D**_**t**_ tensor in the model. The methodology of DTI-based tensor calibration and tissue segmentation to build a 3D computational model for CED has been reported in detail elsewhere [22, 23, 33]. The studies show that within the gray matter, diffusivity and hydraulic conductivity are nearly identical in all directions [2, 22]. Indeed, the assumption of an isotropic target based on published DTI data has been used in the present model, and **D**_**t**_ was approximated by a constant value. On the other hand, the studies show that in the white matter, the bulk flow is preferential along the fiber tracts [2, 23]; consequently, the tensors should project the anisotropy of the target in the model. In the case of complex structures, such as the striatum, a voxel-by-voxel segmentation of target and surroundings into either the gray or white matter should provide more accurate predictions for *V*_d_. The intrinsic tissue transport properties may also be altered because of edema and tissue deformation caused by CED. In the present model, these effects were accounted for using an increased value of porosity *ϕ*, while in normal tissue, *ϕ* = 0.2 [21]. We expect to account for anisotropy of these effects in the 3D model as well.

The numerical limitation of the present model is an inability to simulate the distribution within the catheter surrounding due to the high calculated velocity *v*_*r*_ immediately after the fluid exits the catheter. The missing volume was calculated as 0.03 mL. Since the model was applied to study the clinical application of CED with a projected *V*_d_ of 0.5 mL, this numerical limitation was not meaningful. Meanwhile, examples of CED models for small-volume simulations (e.g., in rodents [22, 23]) demonstrate that this limitation can be overcome with a computational fluid dynamic software approach.

In conclusion, several aspects of IPu delivery of r-metHuGDNF have been studied by means of modeling. The results confirm the slow elimination of GDNF from the striatum with a half-life of about 19 h and suggest that local ISF flow is the primary clearance mechanism. The modeling predicts that a cylindrical pattern of distribution is more favorable for the posterior dorsal putamen, increasing coverage and reducing the risk of distribution outside of the region of interest. No significant differences in distribution were found between infusion rates of 3 and 5 μL/min; both CED rates result in rapid and efficient r-metHuGDNF distribution. Finally, because of its smaller molecular weight, and therefore its greater effective diffusion, clinical-grade Gd-DTPA (Magnevist®) overpredicts the distribution volume of GDNF especially when imaging is performed post-infusion.
